# Inbound medical tourism to Barbados: a qualitative examination of local lawyers’ prospective legal and regulatory concerns

**DOI:** 10.1186/s12913-015-0948-3

**Published:** 2015-07-28

**Authors:** Valorie A. Crooks, I. Glenn Cohen, Krystyna Adams, Rebecca Whitmore, Jeffrey Morgan

**Affiliations:** Department of Geography, Simon Fraser University, 8888 University Drive, Burnaby, BC V5A 1S6 Canada; Harvard Law School, Harvard University, 1563 Massachusetts Avenue, Cambridge, MA USA; Faculty of Health Sciences, Simon Fraser University, 8888 University Drive, Burnaby, Canada

**Keywords:** Medical tourism, Legal, Barbados, Qualitative, Regulation

## Abstract

**Background:**

Enabled by globalizing processes such as trade liberalization, medical tourism is a practice that involves patients’ intentional travel to privately obtain medical care in another country. Empirical legal research on this issue is limited and seldom based on the perspectives of destination countries receiving medical tourists. We consulted with diverse lawyers from across Barbados to explore their views on the prospective legal and regulatory implications of the developing medical tourism industry in the country.

**Methods:**

We held a focus group in February 2014 in Barbados with lawyers from across the country. Nine lawyers with diverse legal backgrounds participated. Focus group moderators summarized the study objective and engaged participants in identifying the local implications of medical tourism and the anticipated legal and regulatory concerns. The focus group was transcribed verbatim and analyzed thematically.

**Results:**

Five dominant legal and regulatory themes were identified through analysis: (1) liability; (2) immigration law; (3) physician licensing; (4) corporate ownership; and (5) reputational protection.

**Conclusions:**

Two predominant legal and ethical concerns associated with medical tourism in Barbados were raised by participants and are reflected in the literature: the ability of medical tourists to recover medical malpractice for adverse events; and the effects of medical tourism on access to health care in the destination country. However, the participants also identified several topics that have received much less attention in the legal and ethical literature. Overall this analysis reveals that lawyers, at least in Barbados, have an important role to play in the medical tourism sector beyond litigation – particularly in transactional and gatekeeper capacities. It remains to be seen whether these findings are specific to the ecology of Barbados or can be extrapolated to the legal climate of other medical tourism destination countries.

## Background

When patients cross national borders to access medical procedures outside of formal cross-border care arrangements they are participating in medical tourism [1]. Medical tourism is a global health services practice enabled by globalizing processes such as trade liberalization that, according to industry reports, is growing [2–5]. Patients visit medical tourism hospitals and clinics for a full range of procedures, including unproven procedures (e.g., stem cell therapies) and ones that are illegal or unavailable in their home countries [6–8]. Medical tourists purchase care abroad for a number of reasons, including having been assigned to lengthy wait lists at home, wanting to obtain a treatment or procedure that is not readily available at home, and identifying lower-cost care abroad [9].

A growing number of countries are developing medical tourism sectors because they want to strengthen local health systems, retain health workers, and/or gain access to revenues [1, 2, 10]. Some destination countries have well established medical tourism clinics and hospitals, such as India and Thailand, while other countries are in the nascent stages of sector development, such as several Caribbean nations. Barbados, a small English-speaking nation in the Lesser Antilles island chain in the Caribbean, is one such country and serves as the focus of this article.

While Barbados has long exported health services to residents of surrounding countries through various cross-border care arrangements, the country is now looking to establish itself as a future destination for international medical tourists. Since the early 2000s initiatives and activities have been undertaken in order to create momentum for sector development. In 2002, a locally owned fertility centre that regularly attracts international patients from the United States (US), United Kingdom, Canada, and throughout the Caribbean was opened. In 2009, the national government began to accept bids to revitalize a derelict public hospital for private purposes, with the goal of developing a purpose-built medical tourism hospital [11]. It awarded a 25-year lease to American World Clinics, a US-based firm with plans to open a 50-bed multi-specialty hospital primarily staffed by international physicians and aimed at treating international patients [11]. To date, this project has encountered numerous delays and has yet to break ground. There are also talks of expanding or renovating the country’s only public hospital, Queen Elizabeth Hospital, in order to create new capacity for treating medical tourists [11–13]. This plan has also yet to be acted upon. In sum, Barbados’ medical tourism sector is currently very small, consisting mainly of the fertility centre, while there is a desire held by many local stakeholders for it to expand. We have elsewhere published a very detailed overview of Barbados’ nascent medical tourism sector that offers much more detailed insight and can be turned to for more information on the local context, including its health system and tourism sector [11].

Facilities providing care to medical tourists do not operate in a vacuum. Instead, these hospitals and clinics interact directly and indirectly with numerous structures, such as zoning and local by-laws, and systems, such as public health care systems, in destination countries. Medical tourism scholars have rightfully pointed out that this practice also interacts with local legal systems in destination countries such as Barbados. Two significant issues have been repeatedly raised about these interactions. First, there is concern as to whether or not patients who travel abroad and are injured in their course of care will face obstacles in suing for medical malpractice in destination nations [14–16]. A particular worry is that destination country legal systems will be less remunerative than the ones in medical tourists’ home countries [14, 15]. Second, there is uncertainty about the effect of medical tourism on destination country public and private health care systems and the efficacy of trade law, law and development, and intergovernmental organizations as potential forms of regulation in order to mitigate negative impacts on these systems [10, 17, 18]. These two concerns are not exhaustive, but they do predominate. Because medical tourism interacts in very direct ways with the legal systems of destination nations, lawyers in these countries are effectively positioned as stakeholders in the medical tourism sector. Meanwhile, the scholarly literature on medical tourism rarely positions them as such, nor considers their perspectives directly.

While the existing legal literature about medical tourism raises many important issues that warrant serious consideration, very little of this research is empirical or based on first-hand insights from destination countries. In this article we take a step towards addressing this knowledge gap by examining the prospective legal and regulatory concerns identified by lawyers in Barbados, an emerging medical tourism destination nation. Specifically, we draw on the findings of a focus group conducted in 2014 with a diverse group of Barbadian lawyers to identify key concerns they have with the intended development of an expanded medical tourism sector in their country. We offer what we consider to be a prospective account because the country’s medical tourism sector is nascent, and so the lawyers spoke primarily to *anticipated* legal issues associated with its development during the focus group. Presenting the findings of a thematic analysis, we show that the Barbadian lawyers we spoke with identified five main legal and regulatory issues. In the discussion section we consider how these issues relate to the existing legal scholarship on medical tourism and also how they speak to the diverse roles that lawyers in destinations have as stakeholders in medical tourism.

We believe this analysis makes a novel contribution to the international medical tourism literature though its direct engagement with lawyers in an emerging destination nation, while also contributing to the small but growing literature on the development of medical tourism in Caribbean countries e.g., [19–22]. As this is a qualitative study, our goal is not to produce generalizable results that can be seen as applying to the legal systems of all nascent medical tourism destination nations, or even simply those in the Caribbean. Instead, in keeping with the qualitative paradigm, we seek to provide adequate context for the findings so that others can determine whether or not the legal and regulatory concerns identified herein are applicable to other countries with similar contexts – something known as seeking transferability [23].

## Methods

The objective of this exploratory qualitative study was to gather empirical insight into lawyers’ prospective concerns about medical tourism in a country seeking sector development. We focused on Barbados for two reasons. First, because it is a small country – having a total population of just over 280,000 – we believed that we would be able to adequately capture the full scope of legal and regulatory concerns held by local lawyers using the focus group method while gaining a good understanding of the relevant local context. Second, we are conducting ongoing research about the health equity impacts of the development of medical tourism in Barbados [11, 24], and so knew we would be able to draw on our local networks to aid in recruitment. To accomplish our objective we, a team of medical tourism and health law experts, held a focus group with lawyers from across the island at a professional meeting venue in Bridgetown, Barbados in February, 2014.

### Recruitment

After obtaining ethical approvals from the Office of Research Ethics at Simon Fraser University, University of the West Indies-Cave Hill/Barbados Ministry of Health Research Ethics Committee/Institutional Review Board, and the Committee on the Use of Human Subjects of the Institutional Review Board at Harvard University, we sent letters of information about the study to members of our local networks as well as local legal societies. The Dean of the local law school agreed to circulate the letter to school members and associates. We hand-delivered letters to law offices and also sent letters to those who had self-identified as Barbadian lawyers on publicly accessible social networking sites. Interested individuals were asked to contact a member of our team to obtain more information about the study and express an interest in participating.

Our goal was to recruit 8–12 participants with diverse legal backgrounds for a single focus group. We had direct contact with 21 potential participants. These potential participants were informed about the focus group time and location and told that because the sector is at a nascent stage and few local lawyers have any expertise on the issue they did not need to have particular familiarity with medical tourism in order to take part in the study. Ten lawyers agreed to participate in the study, one of whom had to withdraw on the day of the focus group due to a scheduling conflict.

### Data collection

The focus group was held mid-day at a central location in the city of Bridgetown, Barbados. It ran for 2 h and a catered lunch was provided. Two investigators co-moderated the discussion while a third member of the team served as note taker. After consent forms were signed by all participants the focus group started with a round of introductions. Next, the co-moderators summarized the study objective and meeting structure and asked participants to share what they knew about medical tourism in Barbados. For the remainder of the focus group the moderators engaged participants in identifying the local implications of medical tourism sector development in the country and the anticipated legal and regulatory concerns via unstructured discussion. At times the moderators sought clarification or invited deeper discussion of particular concerns, but they did not introduce new concerns as we wanted only to investigate those organically raised by the participants. Upon completion of the focus group, participants were given a small gift (a box of locally-made chocolates) to acknowledge their valuable contributions to knowledge.

### Data analysis

The focus group was digitally recorded and transcribed verbatim. We each independently reviewed the transcript to identify emerging themes, a process consistent with thematic analysis. Following this, we met in order to discuss the themes we had independently identified and seek consensus on the scope and interpretation of dominant themes related to legal and regulatory concerns. Five such dominant themes were identified. After reaching consensus, the transcript was hand-reviewed in order to extract data relevant to each dominant theme and identify quotes suitable for inclusion here. We next compared these themes to the existing legal literature about medical tourism to identify points of confirmation and divergence in order to frame our analysis and discussion.

## Results

Nine lawyers participated in the focus group, 6 women and 3 men. They trained for their law degrees in Trinidad, Jamaica, Barbados, England, and Scotland. They held academic appointments in law programs or had practiced law for between 9 and 37 years in firms of various sizes, specializing in areas such as offshore banking, personal injury litigation, business transactions, and medical malpractice. None of the participants had specific expertise in medical tourism, but drew on relevant experience (e.g., working with international investors, representing clients in medical malpractice suits) and their knowledge of national law and the local legal culture to speak to the issues at hand. Participants were generally supportive of the development of the medical tourism sector in Barbados, but their comments displayed an interest in cautiously doing so in a way that would maximize local benefits. Five dominant legal themes arose in the focus group discussion: (1) liability; (2) immigration law; (3) physician licensing; (4) corporate ownership; and (5) reputational protection. In this section we discuss each separately.

### Liability

Perhaps not surprisingly, participants raised legal liability regarding medical malpractice as a main concern. One participant drew an explicit comparison between the likely experience medical tourists from North America would have suing for medical malpractice in Barbados as opposed to at home: “*The [monetary] award is not gonna be as high obviously as if it were in America or in Canada. Secondly the delay [in waiting for a judgment] is gonna be long (laughter). Court here moves slowly. Thirdly, the decision will more than likely be a well-reasoned decision.*” Participants emphasized the way local relationships on the island helped mitigate these downsides. For example, in medical malpractice litigation involving Barbadian patients and Barbadian physicians, several participants explained that settlement was the norm because of the “*medical defense union and their a team of lawyers and quite often they advise doctors who are facing this to settle so very often it doesn’t even get to the courts.”* Participants anticipated that the same would be true in cases involving medical tourists. They also emphasized the fact that most Barbadians liked their physicians and thought highly of them, which helped to lessen the propensity to pursue litigation. They were unsure as to whether or not medical tourists would feel the same way.

Questions were raised by participants as to how both foreign patients and foreign physicians would fit into Barbados’ cordial, settlement-oriented malpractice system. Would foreign physicians be able to take advantage of the medical defense union? Would foreign patients be willing to settle, lacking the sense of bonhomie with the professionals the locals have? Though important, these questions currently remain unanswered given the nascent stage of medical tourism sector development in Barbados. Meanwhile, all participants agreed that if medical tourists needed to pursue trial for medical malpractice, they would face obstacles. Time commitment was thought to be the most significant obstacle. For example, one participant mentioned a medical malpractice case that was ongoing after three years and pointed to the difficulty of getting expert testimony since most Barbadian physicians did not want to testify against their local colleagues. Participants felt that although there were likely to be obstacles associated with dealing with medical malpractice suits involving international patients, Barbados’ sound legal reputation served as a strength in recruiting medical tourists, unlike in other countries where “*something goes wrong with some medical procedure and at the end of the day the jurisdiction is being pointed to as some back-office operation that pretty much would’ve allowed and facilitated this injurious activity to take place.”*

### Immigration law

Participants raised concerns regarding immigration processes in relation to developing an expanded medical tourism sector in Barbados, pointing out that certain aspects of immigration law should at least be reviewed if not even amended in order to facilitate the movement of involved groups into and out of the country. Their concerns pertained to two specific groups: international physicians coming to work at medical tourism facilities and international patients travelling for treatment. Regarding the former, a participant explained that “…*in terms of bringing doctors into Barbados…that’s a big consideration when you were thinking of what the things to consider [are] and do you have to put in any additional regulation. Because regulatory components are important…in terms of being able to live and work in Barbados*.” Participants agreed that if the medical tourism sector was expanded to include international physicians practicing locally, and especially for short periods, there would need to be a review of immigration law to ensure that laws and procedures did not serve as significant barriers to their entry or continued re-entry.

There was awareness among participants that immigration laws for visitors to Barbados can, at times, be “*problematic*” and “*quite difficult to deal with*.” As such, it was not surprising that participants were concerned about the potential difficulties that international patients may face in relation this regard. They were especially concerned about cases where patients may need to extend their stays beyond their approved visa period, which could happen in the event that a medical tourist experiences a complication requiring further treatment that prevents safe travel home. One participant relayed an example of a visitor who had to extend her visa after arrival because she required unplanned medical care and “*it got to the point where she had to go to the court and get a visa extension…[with medical] records that said she couldn’t travel because she needed to rest*.” Participants agreed that the process of appealing for visa extensions can be stressful, and expected that this stress would be amplified in the case of medical tourists who would have to navigate this process while recovering from procedures.

### Physician licensing

Suggesting that an expanded medical tourism sector in Barbados would need to operate within a *“proper structure”*, participants highlighted physician licensing as one mechanism for ensuring this structure was in place. This concern pertained specifically to the temporary migration of foreign health workers to medical tourism facilities, and especially for short periods of time (wherein existing licensing procedures focus primarily on foreign trained health workers permanently relocating to Barbados). While participants noted that the process of licensing foreign physicians for local practice as a matter of health law might face onerous *“red tape”*, it was also framed as an opportunity to *“put everything in place”* and ensure international physicians meet Barbadian licensing standards. Participants were adamant that Barbadian licensing standards be maintained as the gold standard for local practice instead of allowing for equivalencies. They thought that Barbadian lawyers were positioned to *“explain how the licensing and registration have to comply with laws”* to those parties interested in developing new medical tourism facilities, and especially international investors.

Participants pointed out that the practicalities of developing licensing mechanisms for international physicians working at medical tourism facilities were likely to be challenged by the fact that such individuals would not be members of national health worker unions. They questioned the potential for even developing acceptable licensing for foreign physicians, as well as medical malpractice coverage for their practices, without union affiliation: “*so these doctors…are not going to be part of the union, right? So whether the [malpractice] litigation [process] will go differently…or whether you’re anticipating that in order to practice here you will at least provisionally have to be a member of the union.”* This issue demonstrates that considerations of international physician licensing as it relates to developing the *“proper structure”* of medical tourism are complicated by the structures already in place to regulate health workers in the country. Participants cautioned that these structures, including existing practices around union membership, should not be altered to the detriment of local health workers and Barbadian patients in order to accommodate an expanded medical tourism sector.

### Corporate ownership

Participants spent significant time discussing the potential for corporate structure and ownership, and especially international ownership, of medical tourism facilities in Barbados. There was a strong sense that if things were set up correctly to begin with, many of the other legal and regulatory concerns they identified could be handled or mitigated. Conversely, the wrong corporate structure and ownership could be disastrous both for the medical tourism sector and the island’s reputation wider reputation in the international community. As one participant put it: “*if someone is going for medical tourism reasons, they definitely don’t want to go to somewhere that has been undercapitalised because you don’t want to hear that in the middle of [a procedure]…the place is closing… [It must be] well capitalised and well managed. So I think we can start by saying we have an example here of the best of medical tourism, and that is with the [fertility clinic].”* Participants believed that good regulation of corporate structure and ownership could guard against predatory clinics and those with bad business practices from being established on the island.

The issue of investment capitalization and security was explicitly tied, by participants, to the extent of foreign ownership of medical tourism facilities and registration requirements for these investors. One participant explicitly drew comparisons with foreign investments in other sectors of Barbados’ economy and offered three possible models for how investment in the country’s medical tourism sector could unfold:

*There is a history of foreign investment into Barbados: [first] registering with the central bank of Barbados, [next] coming to the appropriate arrangements…there’s models of it working. The first model is the one I mentioned off the bat [referring to the fertility centre]…that is as close to perfection as you get because it’s a local investment… [Second] you have the next best, which is the venture where foreign investment teams up with a local person to do something. Or [third] you can get pure foreign investment where investors just come in, and even that is good because they’re generating employment, they’re bringing in capital… So if you look at all three models there are significant national benefits if they are properly done. And the laws are there to protect all three.*

In each of these investment arrangements, local Barbadian lawyers would need to assist with ensuring that the best interests of investors and of local citizens and health workers alike were equitably balanced in an expanded medical tourism sector. Participants pointed out that doing so requires legal knowledge relating to property acquisition, environmental protection, and health professional licensing and insurance.

### Reputational protection

Participants expressed concern that Barbados’ reputation as a safe destination for tourists might be compromised due to the legal and regulatory concerns shared above. One participant said that particularly in the case of foreign-owned hospitals or clinics, “*there’s a question for the host jurisdiction in terms of the reputational risk.*” For example, should a malpractice or immigration decision negatively impact a medical tourist, subsequent media attention could negatively impact Barbados’ medical tourism brand as a whole. Similarly, Barbados could receive negative press if licensing issues arose or if a poorly-managed clinic was operating. For example, if “*something goes wrong with some medical procedure and at the end of the day the jurisdiction is being pointed to as some back-office operation,*” that could harm Barbados’ reputation as a safe place for tourists. A past example of an unlicensed stem cell clinic seeking to treat international patients operating in Barbados for a brief period was raised. It was noted that this clinic was shut down by the government and received international attention. As a result of the reputational risk posed by medical tourism, participants thought it would be reasonable for Barbados to require future clinics to have a certain level of “*transparency*” regarding their oversight and regulation.

Because of the legal and regulatory risks that scaling up the medical tourism industry might pose, participants suggested that local lawyers are “*gatekeepers*” who are responsible for protecting the reputation or brand of Barbados. Overall, participants agreed that the Barbadian government and lawyers would need to do their “*due diligence*” and investigate potential medical tourism developments with care. They articulated the need for lawyers to structure and regulate medical tourism developments in order to protect Barbados and maximize the benefits to the country. In their view:

*…the use of the word red tape is not necessarily derogatory. The reason why people have confidence in the Barbados product is that we know that once it is put together, there is trust in it. There is integrity to it. So even if you bring in an overseas clientele, it’s like “yeah, I know this is in Barbados, but they are well-regulated, I trust the product that is coming.”*

The general sentiment that Barbados has a strong tourism brand that depends on high safety standards and robust regulation was shared by all participants. This perspective emphasized the need for lawyers to guard against harms being imposed by an expanded medical tourism sector that would threaten the Barbados brand.

## Discussion

The findings show that clear legal and regulatory tensions were evident in participants’ prospective views of medical tourism in Barbados. While they were generally supportive of the expansion of medical tourism, their interest in developing this sector in the ‘right’ way was stymied by tensions around how to balance various interests and ideas, including: the need for rigorous regulation with a welcoming environment for business; the pros and cons of local and/or foreign ownership; and, at times, the competing interests of local and foreign health workers. In particular, discussion of “red tape” raises interesting questions about how Barbados’ established tourism reputation can be protected in light of medical tourism sector expansion and how this can occur in the best interests of all Barbadians. Debate over the different business models for investment in medical tourism and concern for the opinions of local medical professionals highlighted the importance participants placed on developing a medical tourism industry that will benefit Barbados and centrally involve local lawyers as key stakeholders. Generally, the participants perceived the resolution of these tensions as central to ensuring the viability of the medical tourism sector in Barbados and to gaining public buy-in for its further development.

As lawyers, participants focused their discussion on the potential impacts of the medical tourism on Barbadian law and regulatory structures, as well as the capacity of the existing legal structure to protect the country while navigating the tensions that emerge in medical tourism planning. The points they raised are reflective of many of the ethical and legal considerations raised in the existing medical tourism literature. In particular, concerns for liability due to malpractice are extensively discussed in this literature. Much of this discussion pertains to the complexity of determining jurisdiction in such cases given the international dimension of care, and whether or not international patients are protected by local malpractice regulations [14–16]. This same concern also organically emerged in the focus group discussion, empirically demonstrating that lawyers in medical tourism destination nations are indeed challenged with understanding if and how international patients are protected against malpractice by their local laws. Furthermore, participants’ identification of the value of pursuing this industry despite the complex legal and ethical considerations are in line with the commonly cited benefits of the medical tourism industry [6–8]. Participants hoped for sector expansion to bring new employment opportunities and investment revenues into the country. Given the nascent stage of Barbados’ medical tourism sector, it will be many years before these benefits may be realized and for it to be determined whether or not such benefits outweigh the practical outcomes of the legal and regulatory concerns identified by this study.

Some of the legal and regulatory concerns identified by participants offer new insights into the issues faced by the development of medical tourism in destination countries. Specifically, there is little existing recognition of: the potential for local pushback to foreign investment; the role of ownership structures in providing solutions to concerns over medical tourism development; the implications for immigration law; and local concerns for protecting a country’s reputation and tourism brand. While the literature does highlight the interplay between private medical tourism facilities and public health care systems in destination countries [10, 17, 18], given the findings of this study, it is perhaps surprising that the interplay between systems such as immigration law and the desire to hire foreign health workers and local investment regulations and the desire to attract international investors are not featured more prominently in the legal research about medical tourism. Furthermore, the focus group findings emphasize the need for regulation and policy that ensures Barbadians benefit adequately from ownership of future medical tourism facilities. Participants’ interest in discussing the structure and style of such ownership was unexpected as typically the literature, and especially that focused on health equity, presents a less optimistic view of international ownership in particular [10, 17, 18]. Participants’ interest in examining ownership models may be informed by the Barbadian context, in which foreign investment is typically relied upon for capital-intensive industry development [25], like that associated with medical tourism. However, discussions about foreign investment in medical tourism do not commonly distinguish between differing contexts to constructively provide recommendations for ensuring that desired benefits from medical tourism are optimized in destination countries [26].

The findings paint a rich picture of the roles lawyers play as medical tourism stakeholders, and confirm our earlier assertion that lawyers must indeed be positioned as stakeholders in this sector. Given that lawyers have a professional interest in obtaining and maintaining employment, there is the possibility that participants emphasized the breadth of roles that they could play in Barbados’ nascent medical tourism sector with this in mind. This said, much of the existing legal scholarship on medical tourism considers lawyers in their role as litigators, in particular due to the focus on medical malpractice litigation and the ways in which medical tourism inflects such litigation [14–16]. While participants discussed litigation, for the most part they viewed lawyers as playing several additional roles, roles they emphasized much more than the litigator role. Participants spent much of their time discussing a more transactional role for lawyers: helping to set up deals, helping to advise clients how to choose appropriate foreign partners and when to avoid entering the medical tourism sector altogether, and generally troubleshooting potential obstacles for a successful business. They spoke extensively about topics such as capitalization, registration with the bank of Barbados, ensuring the proper channels for immigration, and the like. Participants also emphasized lawyers’ roles as “gatekeepers” responsible for protecting the reputation or brand of Barbados above and beyond what might be the interests of their clients. This role was unexpected and also offers a novel contribution to the legal literature on medical tourism. Participants held the regulatory structures that guard other forms of investment on the island and its tourism industry in high regard and saw themselves as having a role in getting it “right” so as to not bring disrepute to the island’s outreach efforts.

Future research could usefully examine whether the lawyers in other established and emerging destination countries in and outside of the Caribbean have a similarly nuanced conception of their roles along the lines of what was found in the current analysis. For example, such research could ask: do lawyers practicing in countries with very different medical malpractice and/or international investment contexts view their roles in their own local developing medical tourism sectors similar to how lawyers in Barbados do? It would also be useful to learn how much of this dynamic resulted from elements specific to Barbados’ legal culture – such as a relatively small geography and small legal community, host country of the regional law school, cordial relations between lawyers and physicians reported by participants, high settlement rate for medical malpractice also reported by participants – or whether such dynamics appear in very different legal cultures as well. Undertaking similar research in other countries is the only way to know how much Barbados’ legal culture shaped the findings reported on here. No doubt the legal cultures of other countries will shaped by very different elements, but we currently do not know if these different cultures have an impact on the scope of roles that lawyers can and do play in their local medical tourism sectors. Such comparative knowledge will aid in identifying potential constraints on the transferability of the findings of this analysis to other jurisdictions in the wider Caribbean and beyond.

### Study limitations

We have two main limitations to report. First, we acknowledge that the full scope of legal expertise relevant to the topic at hand was not represented in the focus group. For example, none of the lawyers had specific expertise in the hospitality and tourism sector, representing health worker organizations, or working for public or private health care entities. We cannot say with any certainty whether such perspectives would have altered the five themes identified and the analysis presented here. Second, a limitation of all focus groups is that some participants may dominate the discussion more than others [27]. This was observed in our focus group, wherein one prominent and well-regarded participant spoke most often. To counter this we actively invited discussion from other participants.

## Conclusions

This study represents the first examination of legal and regulatory concerns associated with expansion of medical tourism in Barbados, and indeed one of the first attempts to study the prospective views of destination country lawyers about medical tourism anywhere in the world. The lawyers we spoke with expressed opinions on two legal and ethical issues that have received predominant attention in the literature: the ability of medical tourists to recover medical malpractice for adverse events and the effect of medical tourism on access to health care in the destination country. Participants also identified several topics that have received much less attention in the legal and ethical literature: immigration issues facing medical tourist patients and physicians who come to practice temporarily at facilities; licensing of foreign physicians who come to practice temporarily; corporate structure, ownership, and capitalization; and the role of lawyers in protecting the destination country’s reputation. More generally, this exploratory analysis shows that at least for Barbados, lawyers have an important role to play in the medical tourism sector beyond litigation, acting in transactional and gatekeeper capacities much more than the existing literature suggests. It remains to be seen whether focus groups of lawyers in other jurisdictions would show similar results, or whether there are specific aspects of the regulatory ecology of Barbados that make the Barbados experience distinctive. We call on researchers to undertake studies in jurisdictions that do not share this same regulatory ecology in order to assess the ways in which the findings presented herein carry forward to other countries looking to establish medical tourism sectors.

## Abbreviation

US: United States
